# Identification of Transcription Factors Involved in the Regulation of Flowering in *Adonis Amurensis* Through Combined RNA-seq Transcriptomics and iTRAQ Proteomics

**DOI:** 10.3390/genes10040305

**Published:** 2019-04-18

**Authors:** Aimin Zhou, Hongwei Sun, Shengyue Dai, Shuang Feng, Jinzhu Zhang, Shufang Gong, Jingang Wang

**Affiliations:** 1 College of Horticulture and Landscape Architecture, Northeast Agricultural University, Harbin 150030, China; aiminzhou@neau.edu.cn (A.Z.); sunhongwei0720@163.com (H.S.); 15246468669@163.com (S.D.); jinzhuzhang@neau.edu.cn (J.Z.); 2 Key Laboratory of Saline-Alkali Vegetation Ecology Restoration in Oil Field (SAVER), Ministry of Education, Alkali Soil Natural Environmental Science Center (ASNESC), Northeast Forestry University, Harbin 150040, China; fengshuang86@163.com

**Keywords:** *Adonis amurensis*, flowering gene, transcriptome, proteome, transcription factor

## Abstract

Temperature is one of the most important environmental factors affecting flowering in plants. *Adonis amurensis*, a perennial herbaceous flower that blooms in early spring in northeast China where the temperature can drop to −15 °C, is an ideal model for studying the molecular mechanisms of flowering at extremely low temperatures. This study first investigated global gene expression profiles at different developmental stages of flowering in *A. amurensis* by RNA-seq transcriptome and iTRAQ proteomics. Finally, 123 transcription factors (TFs) were detected in both the transcriptome and the proteome. Of these, 66 TFs belonging to 14 families may play a key role in multiple signaling pathways of flowering in *A. amurensis*. The TFs FAR1, PHD, and B3 may be involved in responses to light and temperature, while SCL, SWI/SNF, ARF, and ERF may be involved in the regulation of hormone balance. SPL may regulate the age pathway. Some members of the TCP, ZFP, MYB, WRKY, and bHLH families may be involved in the transcriptional regulation of flowering genes. The MADS-box TFs are the key regulators of flowering in *A. amurensis*. Our results provide a direction for understanding the molecular mechanisms of flowering in *A. amurensis* at low temperatures.

## 1. Introduction

Flowering is the transition from vegetative to reproductive growth and is a key developmental stage of successful seed production in flowering plants [1]. To assure timely flowering in various environments, plants have formed several main genetic mechanisms that are regulated by external and internal signals. Currently, six major pathways are known to control flowering: photoperiod pathway, vernalization pathway, ambient temperature pathway, gibberellin pathway, the autonomous pathway, and age pathway [2]. In the photoperiod, vernalization, and ambient temperature pathways, light, and temperature act as external signals and are the most important environmental factors in the regulation of flowering time [2,3]. The gibberellin, autonomous, and age pathways regulate flowering by responding to internal signals [4,5,6].

In the annual model plant *Arabidopsis thaliana*, some of the major genes that have been shown to affect flowering time have been placed in the flowering pathway [2,7]. In the photoperiod pathway, phytochrome and cryptochrome photoreceptors sense light signals and reset the circadian clock feedback loop, including circadian clock associated 1 (CCA1), late elongated hypocotyl (LHY) and timing of cab expressin 1 (TOC1) [8,9]. Constans (CO) is a transcriptional regulator that plays a key role in photoperiod and the biological clock, and the expression of CO is regulated by day length and the circadian clock. CO promotes flowering by initiating transcription of the flowering genes flowering locus T (FT) and the twin sister of FT (TSF) [8,10,11]. Flowering locus C (FLC) is a MADS-box transcription factor (TF) that acts as a floral repressor and prevents flowering by repressing FT and suppressor of -overexpression of CO 1 (SOC1) [12]. The vernalization pathway promotes flowering by repressing FLC transcription [13,14]. In the vernalization pathway, vernalization 2 (VRN2) and vernalization insensitive 3 (VIN3) are involved in the repression of FLC expression [15,16]. Another MADS-box TF, short vegetative phase (SVP), plays an important role in the ambient temperature pathway. At low temperatures (e.g., 16 °C), SVP prevents flowering by negatively regulating the expression of FT [17]. The autonomous, gibberellin (GA), and age pathways respond to internal signals to regulate flowering. The autonomous pathway promotes flowering by lowering the basal level of FLC transcription [5]. Gibberellin 20 oxidase (GA20ox) is a key catalytic enzyme in the biosynthesis of GA, the concentration of which increases at the meristem immediately prior to floral induction and which promotes flowering by up-regulating SOC1 expression [4]. In the age pathway, concentrations of squamosa promoter binding protein-like (SPL) TFs increase with increasing age and promote flowering by increasing the expression of flowering genes, such as SOC1 and FRUITFULL (FUL). SPLs are negatively regulated by the microRNA miR156 [6,18]. 

In the perennial model plant *Arabis alpina*, *perpetual flowering 1* (*PEP1*) is the ortholog of the *A. thaliana FLC* gene. *PEP1* is transiently repressed by low temperatures, causing repeated seasonal cycles of repression and activation of *PEP1* transcription that allow it to carry out functions characteristic of the cyclical life history of perennials [19]. Moreover, recent studies have shown that *SPL15* regulates flowering of *A. alpina* in response to vernalization [20].

The identification and functional characterization of flowering genes from model plants are useful; however, the identification and characterization of these genes in plants that grow in unique environments and/or under extreme conditions are necessary to further elucidate the molecular mechanism of flowering regulation. *Adonis amurensis* is a perennial herbaceous flower in the family Ranunculaceae. *A. amurensis* can blossom before the ice and snow melts in the spring in northeast China, where the temperature can drop to −15 °C. However, the genetic regulatory mechanisms of flowering in *A. amurensis* under extremely low temperatures are unclear. Currently, both transcriptomics and proteomics have been widely used to identify genes in flowering in plants [21,22,23]. In the present study, we identified the key TFs involved in flowering in *A. amurensis* by combining transcriptomic and proteomic analyses. The findings of our study will aid further understanding of the genetic regulatory mechanisms of flowering plants under extremely low temperatures.

## 2. Materials and Methods

### 2.1. Plant Materials

*Adonis amurensis* plants were grown in an open field at Northeast Agricultural University (Harbin, China; 128.4° E, 45.0° N). The floral organs of *A. amurensis* were sampled simultaneously at different developmental stages and were immediately stored at −80 °C until required for RNA and protein extraction.

### 2.2. RNA-seq and Transcriptome Analyses

Total RNA from the floral organs of *A. amurensis* plants was isolated using TRIzol reagent (Invitrogen, Carlsbad, CA, USA). The quality of the RNA samples was confirmed, and the samples were sent to Bionova Biotech (Beijing, China) for RNA sequencing. cDNA library construction and Illumina sequencing were performed according to the manufacturer’s instructions (Illumina, San Diego, CA, USA). The RNA-seq datasets were deposited in the NCBI Gene Expression Omnibus (GEO) with accession number GSE126456.

Transcriptome data were processed as previously described [24]. The de novo assembly of the *A. amurensis* transcriptome in the absence of a reference genome was accomplished using Trinity. Trinity combines the reads with a certain length of overlap to form longer fragments, known as contigs. These contigs were subjected to sequence clustering to form longer sequences. Such sequences were defined as unigenes. All assembled unigenes were compared with the following public databases: NR (NCBI non-redundant protein sequence), NT (NCBI nucleotide sequence), Swiss-Prot protein, KEGG (Kyoto Encyclopedia of Genes and Genomes), KOG (euKaryotic Ortholog Groups), InterPro, and GO (Gene Ontology) databases.

### 2.3. Analysis of Differentially Expressed Genes (DEGs)

The expression levels of unigenes were calculated using the Fragments Per Kilobase of transcript per million mapped reads (FPKM) were calculated using RSEM [25]. This software calculates the FPKM values for each assembled transcript by normalizing the counts of the paired-end reads to both the length of the transcript and the total number of mapped reads in the sample [26]. The FDR control method [27] was used in multiple hypothesis testing to correct for p values. After the FDR was obtained, the ratio of FPKMs was used to calculate the fold-change in the expression of unigenes in two samples simultaneously. The smaller the FDR and the larger the ratio, the larger was the difference in the expression level between the two samples. In our analysis, DEGs were screened with an FDR threshold of 0.05 or less and an absolute value of the log2 ratio of 1 or more. All DEGs were mapped to each term of the KEGG or GO databases, and significant pathways were defined based on a corrected *p*-value of ≤ 0.05.

### 2.4. Protein Extraction and iTRAQ Proteome Analysis

Protein extraction was performed as previously described [28]. The samples were ground in liquid nitrogen to a fine powder and extracted in lysis buffer, then 1mM phenylmethyl sulfonyl fluoride (PMSF), 2 mM ethylenediamine tetraacetic acid (EDTA), and 10 mM dithiothreitol (DTT) were added. The samples were sonicated for 5 min and centrifuged at 25,000× *g* for 20 min. The supernatant was mixed well with five times its volume of 10% trichloroacetic acid (TCA)/chilled acetone and incubated at −20 °C for 2 h. After centrifugation at 25,000× *g* for 20 min, the supernatant was discarded, and the previous processes were repeated once. The quality of protein samples was confirmed by a Bradford assay and 12% sodium dodecyl sulfate polyacrylamide gel electrophoresis (SDS-PAGE). Samples were subsequently sent to Bionova Biotech (Beijing, China) for proteomic analyses via isobaric tags for relative or absolute quantitation iTRAQ. iTRAQ labeling, MS analysis, and protein identification were performed as previously described [28]. For peptide data analysis, raw data were employed using the Mascot search engine (Matrix Science, London, UK; version 2.3.02). For protein identification, a series of standard parameters were set as follows: 0.1 Da fragmented mass tolerance, 0.05 Da peptide mass tolerance and one maximum missed cleavage.

### 2.5. Quantitative Real-Time PCR (qPCR) for Validation

The expression of three key MADS-box genes and 25 randomly selected genes were investigated by qPCR. The primers for these assays (Table S2) were designed using Primer 5.0, and qPCR was performed using a qTOWER 2.2 PCR system (Analytik Jena, Jena, Germany) and SYBR Green PCR Master Mix (TaKaRa, Dalian, China) according to the manufacturer’s instructions. *AaActin* was used as a reference gene. Three biological and three technical replicates were performed for each sample.

## 3. Results

### 3.1. Transcriptome Sequencing of Floral Organs in A. amurensis

**Figure 1:**
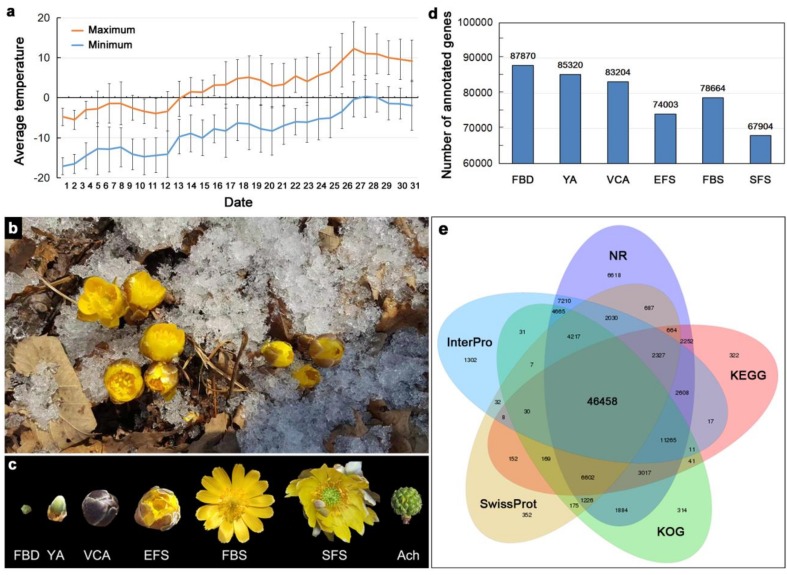
Identification of transcripts in the floral organs of *A. amurensis* at different developmental stages of flowering. (**a**) The mean maximum and minimum temperatures during March in northeastern China in the past 8 years. Error bars indicate SD. (**b**) *A. amurensis* within its range of distribution in northeast China. (**c**) The six stages of floral development in *A. amurensis*. FBD: flower bud differentiation; YA: young alabastrum; VCA: visible color alabastrum; EFS: early flowering stage; FBS: full bloom stage; SFS: senescing flower stage. Ach: achene. (**d**) Number of annotated genes identified in the six floral development stages. (**e**) Venn diagram showing the number of functional annotations for all of the unigenes compared against public databases (NR, KEGG, KOG, Swiss-Prot, and InterPro).

*Adonis amurensis* is a perennial hysteranthous plant with a flowering period of approximately 1 month. At the beginning of March, the flower buds of *A. amurensis* began to sprout, and the lowest temperature at night at this time was approximately −15 °C. *A. amurensis* blooms between March and April in northeast China, when the ice and snow have not melted, and the day and night temperatures are between −15 °C and 10 °C (Figure 1a,b). *A. amurensis* flowers contain approximately nine sepals, initially green, but changing to grayish-purple during flowering, and approximately 10 yellow petals. The flowers open in sunny daylight conditions and close at night to ward off the cold. We divided the flowering process of *A. amurensis* into six developmental stages, including flower bud differentiation (FBD), young alabastrum (YA), visible color alabastrum (VCA), early flowering stage (EFS), full bloom stage (FBS), and senescing flower stage (SFS). To identify genes potentially related to the regulation of flowering in this species, we sequenced the transcriptome of the floral organs of *A. amurensis* at six developmental stages (FBD, YA, VCA, EFS, FBS, and SFS) and compared the results to the following public databases: NR, NT, Swiss-Prot protein, KEGG, KOG, InterPro, and GO (Figure 1c). As a result, a total of 87,870 (FBD), 85,320 (YA), 83,204 (VCA), 74,003 (EFS), 78,664 (FBS), and 67,904 (SFS) genes were annotated in the floral organs of plants sampled from the six developmental stages (Figure 1d and Table 1). Of these genes, 46,458 genes were concomitantly annotated in five of the public databases (Figure 1e). As a result, 39,219 unigenes were successfully assigned to, at least, one GO term, and all resulting GO terms were classified into 53 functional groups in three main categories: biological processes, cellular components, and molecular functions (Figure S1). The majority of annotated genes (40.92%) were similar to those of *Nelumbo nucifera* (Figure S2).

### 3.2. Identification of Differentially Expressed Genes (DEGs) During Flower Development

**Figure 2:**
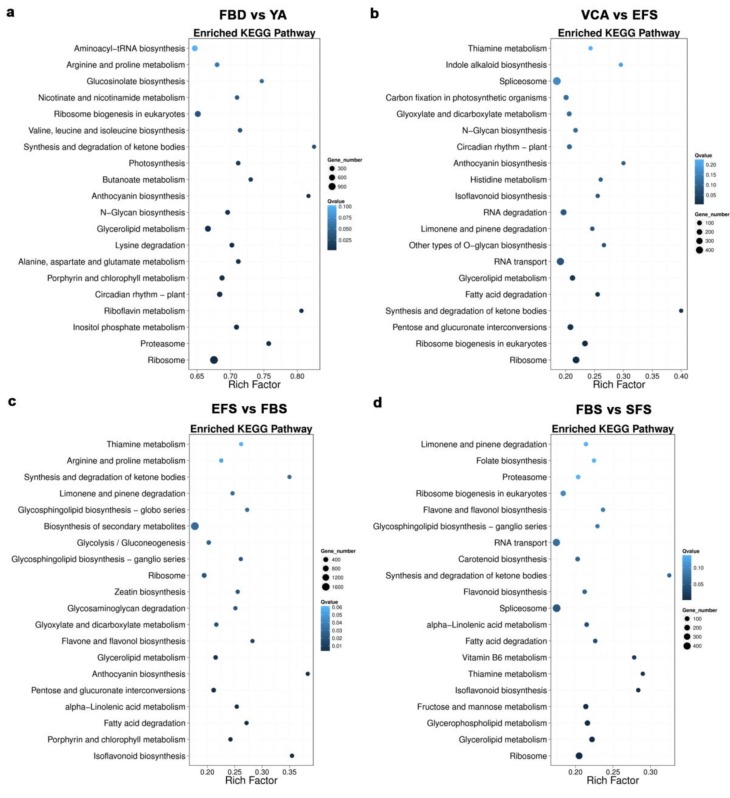
Scatterplot of KEGG pathways enriched for differentially expressed genes (DEGs) in (**a**) FBD vs. YA, (**b**) VCA vs. EFS, (**c**) EFS vs. FBS, and (**d**) FBS vs. SFS in *A. amurensis*. The rich factor is the ratio of the number of DEGs annotated in a given pathway to the number of all genes annotated in the pathway, in which a greater rich factor indicates a greater degree of pathway enrichment. The *Q*-value is the corrected *p*-value and ranges from 0 to 1, where a lower *Q*-value indicates greater intensity. The size of the circles indicates the number of genes in a given pathway. The top 20 enriched pathway terms in the KEGG database are listed.

Using the FPKM method, the DEGs in the floral organs of the six developmental stages were screened. As shown in Figure S3, 37,317 up-regulated and 32,545 down-regulated DEGs were identified in the FBD vs YA comparison group. The numbers of up- and downregulated DEGs were similar (approximately 9000 to 12,000) in the YA vs. VCA, VCA vs. EFS, EFS vs. FBS, and FBS vs. SFS comparison groups. The KEGG pathway enrichment analysis involving the DEGs showed that the anthocyanin biosynthesis and synthesis pathways and degradation of ketone bodies pathway exhibited higher rich factors in several of the comparison groups (FBD vs. YA, VCA vs. EFS, EFS vs. FBS, and FBS vs. SFS), indicating higher degrees of pathway enrichment (Figure 2).

### 3.3. Identification and Classification of Transcription Factors (TF) During Flower Development

**Figure 3:**
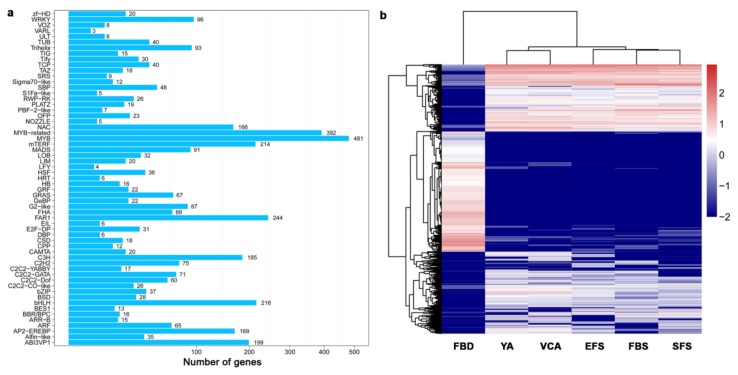
Expression-pattern clustering of transcription factors (TFs) in six floral developmental stages of *A. amurensis*. (**a**) The present study identified 3216 TFs that were classified into 59 categories. (**b**) Expression-pattern clustering of 3216 TFs in floral organs at six developmental stages. The levels of expression of each gene (log2 FPKM) during FBD, YA, VCA, EFS, FBS, and SFS are indicated by red/blue rectangles, where red rectangles represent the up-regulation of genes, while blue rectangles represent down-regulation.

TFs play an important role in the regulation of flowering in plants. Our study identified a total of 3216 TFs and classified them into 59 families via an alignment of conserved domains. The MYB, FAR1, bHLH, mTERF, and ABI3VP1 families of TFs contained the highest numbers of genes (Figure 3a). Clustering analysis showed that the expression patterns of most TFs in FBD were entirely different from the other five stages (Figure 3b). At the FBD stage, members of the MYB, mTERF, FAR1, and bHLH families were mainly up-regulated, and members of the MYB, FAR1, AP2-EREBP, and NAC families were mainly up-regulated at the other five stages.

### 3.4. Proteome Sequencing of Floral Organs in A. amurensis

To more accurately identify the TFs involved in the regulation of flowering, we sequenced the proteome of floral organs at three development stages (YA, EFS, and SFS) using transcriptome data as a reference. Based on LC-MS/MS analyses, a total of 368,236 spectra were generated, which included 83,842 matched spectra and 68,563 unique spectra. In total, 41,938 peptides containing 36,250 unique peptides and 8427 proteins were identified (Table 2). All of the annotated proteins were successfully assigned to at least one of the 52 GO terms, and were grouped into three main categories: biological processes, cellular components, and molecular functions (Figure S4).

### 3.5. Identification of TFs Involved in Flowering by Combining Transcriptomic and Proteomic Data

**Figure 4:**
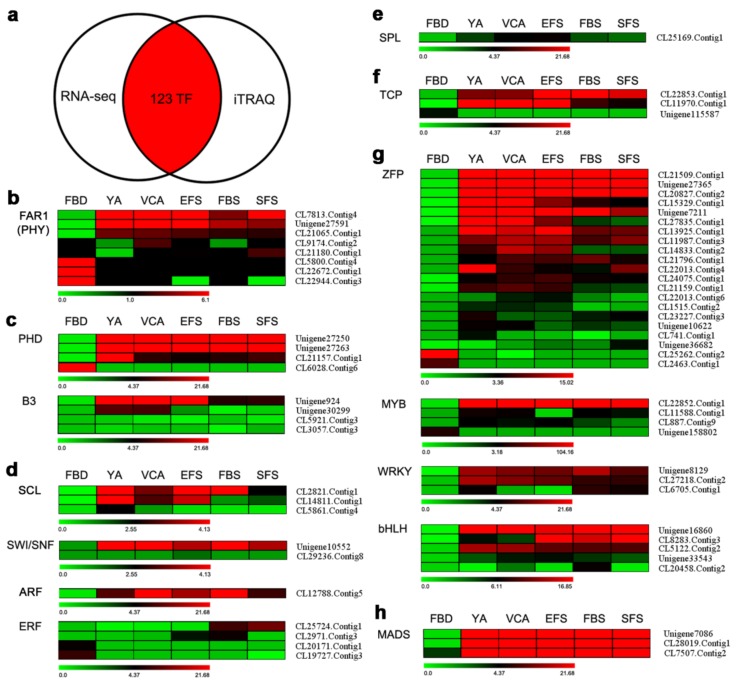
The identification of key transcription factors (TFs) related to flowering via a combination of RNA-seq transcriptomics and iTRAQ proteomics in *A. amurensis*. (**a**) Venn diagram showing the number of TFs that appear in both the RNA-seq transcriptome and the iTRAQ proteomic data. (**b**–**h**) Heatmaps of 66 TFs in flower tissues at six developmental stages. The 66 TFs are grouped into 14 main categories. The bars below each heatmap represent the scale of the expression levels of each gene (FPKM) in the FBD, YA, VCA, EFS, FBS, and SFS stages as indicated by red/green rectangles. Red rectangles represent the up-regulation of genes, while green rectangles represent down-regulation. Information for each gene listed can be found in Table S1.

A combined analysis showed that 123 TFs appeared in both transcriptomic and proteomic data (Figure 4a). Of these TFs, 14 families containing 66 TFs were homologous to the TFs that have been reported to be involved in the regulation of flowering. Of the eight TFs identified that belong to the FAR1 family of TFs, three genes were up-regulated, and three genes were down-regulated (Figure 4b). In Arabidopsis, FAR1 is reported to interact with the phytochrome A signaling component PHY3, while phytochrome A is the primary photoreceptor regulating photoperiod pathway [29]. The four TFs belonging to the PHD family of TFs (three up-regulated and one down-regulated), and four B3 TFs (one up-regulated) were also identified (Figure 4c). VRN2 and VIN3, which are involved in the vernalization pathway of flowering, belong to the PHD and B3 families of TFs, respectively [16,30]. The three TFs belonging to the SCL family (two up-regulated), two SWI/SNF TFs (one up-regulated), one up-regulated ARF TF, and four ERF TFs were also identified (Figure 4d). SCL and SWI/SNF are known to be involved in GA signaling [31,32], while ARF and ERF are involved in auxin and ethylene responses, respectively [33,34]. Homologs of one SPL TF and three TCP (two up-regulated) TFs were also detected in the present study (Figure 4e,f). SPL is a key regulator involved in the age pathway [18], and TCP is reported to be involved in the regulation of flowering by regulating the expression of SOC and CO [35,36]. Furthermore, 21 ZFP, four MYB, four WRKY, and five bHLH TFs were identified (Figure 4g). These four types of TFs have been reported to play a role in flowering by regulating the expression of flowering genes [37,38,39,40]. Finally, the present study identified three up-regulated MADS-box TFs (Figure 4h), as the MADS-box TF family plays a crucial role in controlling floral organogenesis and flowering time in plants [41].

### 3.6. Verification of Gene Expression of TFs Involved in Flowering

**Figure 5:**
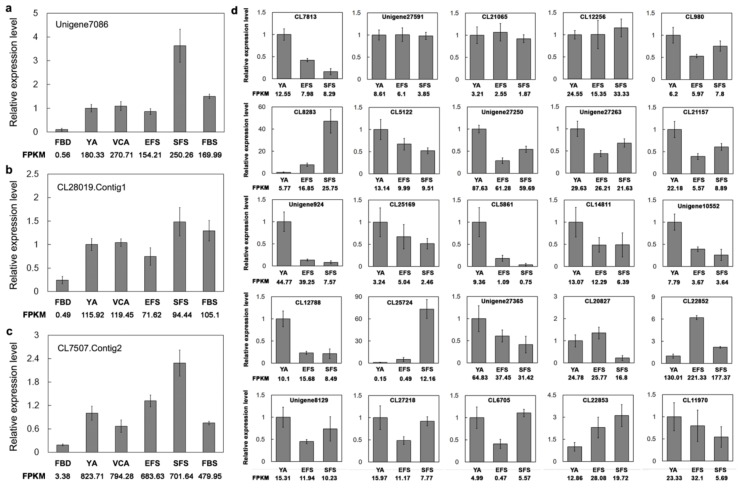
qPCR validation of the expression of key transcription factors (TFs) in *A. amurensis*. (**a**–**c**) The relative expression levels of three MADS TFs during the six developmental stages. (**d**) qPCR analysis of randomly selected 25 TFs. *AaActin* was used as an internal control, and the transcript level in the YA sample was set to 1.0. For each gene, three biological replicates and three technical replicates were performed for each experiment. Error bars indicate *SE*.

We used qPCR to investigate the expression of the TFs identified in the present study. qPCR showed that the three key MADS-box TFs (Unigen7086, CL28019.Contig1, and CL7507.Contig2) were differentially expressed across the six developmental stages of flowering, and also that the highest levels of expression were observed during the SFS stage (Figure 5a–c). In addition, we investigated the expression of 25 randomly selected TFs (three FAR1, three PHD, one B3, two SCL, one SWI/SNF, one ARF, one ERF, two ZFP, one MYB, three WRKY, and two TCP) in the YA, EFS, and SFS stages. Approximately 72% of the TFs were consistent between the qPCR and RNA-seq data (FPKM) (Figure 5d). In general, the qPCR results were consistent with the RNA-seq data, showing that our sequencing data are reliable.

## 4. Discussion

Currently, the transcriptome and proteome have been widely used to study the identification of regulatory genes specific to unique plant traits [42,43]. In our study, a total of 113,899 genes were assembled from the floral organs of *A. amurensis* by transcriptome sequencing, 46,458 of which were annotated in five public databases (Figure 1e). Using the transcriptomic data as a reference, 8427 proteins were identified in the floral organs of *A. amurensis* by sequencing the proteome (Table 2). As a complement to transcriptomic data, proteomic data improves the reliability of sequence identification and the functional annotation of genes. Although TFs are known to play a key role in the regulation of plant flowering, the identification of TFs using proteomic methods is generally limited, as the levels of protein expression of TFs are generally low. In the present study, a total of 123 TFs were identified by combining transcriptomic and proteomic techniques (Figure 4a), indicating that it is feasible to identify TFs in the proteome. Flowering regulation of *A. amurensis* under low temperature may be coregulated by several TFs. A combination of transcriptome and proteome analysis aids in quick and extensive identification of key flowering-related TFs in *A. amurensis*. Our study provides genetic information for the functional characterization of these TFs in the future.

Of all the identified TFs, 66 TFs belonging to 14 families may play a role in the regulation of flowering in *A. amurensis*. FAR1 is known to be involved in the regulation of phytochrome signaling, while 10 CO, five LHY, and one CCA1 TFs were identified from the transcriptome in the present study. Together with FAR1, they may be involved in the photoperiod and circadian clock pathway in flowering of *A. amurensis*. CO, LHY, and CCA1 may not be detected in the proteome owing to their low expression levels. The VINs and VRNs involved in the vernalization pathway of Arabidopsis belong to the PHD and B3 families of TFs, respectively. B3 (Unigene30299) is highly homologous to Arabidopsis VRN1, which plays multiple roles in vernalization and flowering time control [28]. GA act as endogenous signaling molecules to regulate flowering in plants. SCL and SWI/SNF are known to be involved in the GA response, while ARF and ERF have been shown to be involved in auxin and ethylene signaling, respectively. Auxin and ethylene may act synergistically or antagonistically with GA in the regulation of flowering [31,32,33,34]. One SPL identified in the present study (CL25169.Contig1) may be a key regulator of the age pathway in *A. amurensis*. The amino acid sequences of CL25169.Contig1 were 25.9% identical with Arabidopsis SPL12. Three TCP TFs, annotated as TCP4 (CL11970.Contig1), TCP5 (CL22853.Contig1), and TCP11 (Unigene115587), were identified in the present study. In Arabidopsis, TCP4 and TCP5 have been reported to be involved in the development of petals [44,45]. The amino acid sequences of CL11970.Contig1 and CL22853.Contig1 were 50.6% and 20.6% identical with Arabidopsis TCP4 and TCP5, respectively. Four families of TFs, ZFP, MYB, WRKY, and bHLH, have been reported to be involved in the regulation of flowering in several plant species. The CCCH-type ZFP (EHD4) from *Oryza sativa* plays an essential role in the photoperiodic control of flowering time [46], while Arabidopsis C2H2-type ZFP (SUF4) represses flowering via the transcriptional activation of FLC [37]. Expression of the CCCH-type ZFP gene *MsZFN* from *Medicago sativa* delays flowering time in transgenic Arabidopsis [47]. In the present study, 21 ZFPs were identified, including 10 CCCH-type (CL20827.Contig2, Unigene7211, CL2463.Contig1, Unigene27365, CL14833.Contig2, CL21509.Contig1, CL22013.Contig6, CL22013.Contig4, CL13925.Contig1, and CL1515.Contig2) and one C2H2-type ZFP (CL11987. Contig3). The amino acid sequences of CL13925.Contig1 were 40.4% identical with MsZFN. In Arabidopsis, some members of the MYB, WRKY, and bHLH families, such as EARLY FLOWERING MYB PROTEIN (EFM) and FLOWERING LOCUS E (FE) from the MYB family; WRKY12, 13, 71, and 75; and bHLH48 and 60, have been reported to regulate flowering by affecting FT transcription [38,39,40,48]. In our study, four MYB, three WRKY, and five bHLH TFs were identified. Finally, three up-regulated MADS-box TFs (Unigen7086, CL28019.Contig1, and CL7507.Contig2) were identified, which were homologous to pistillata (PI), sepallata 3 (SEP3), and agamous-like 6 (AGL6) from the Arabidopsis MADS family, respectively. It has been shown that PI, SEP3, and AGL6 play key roles in the regulation of floral organ development and flowering time [41,49].

## 5. Conclusions

**Figure 6:**
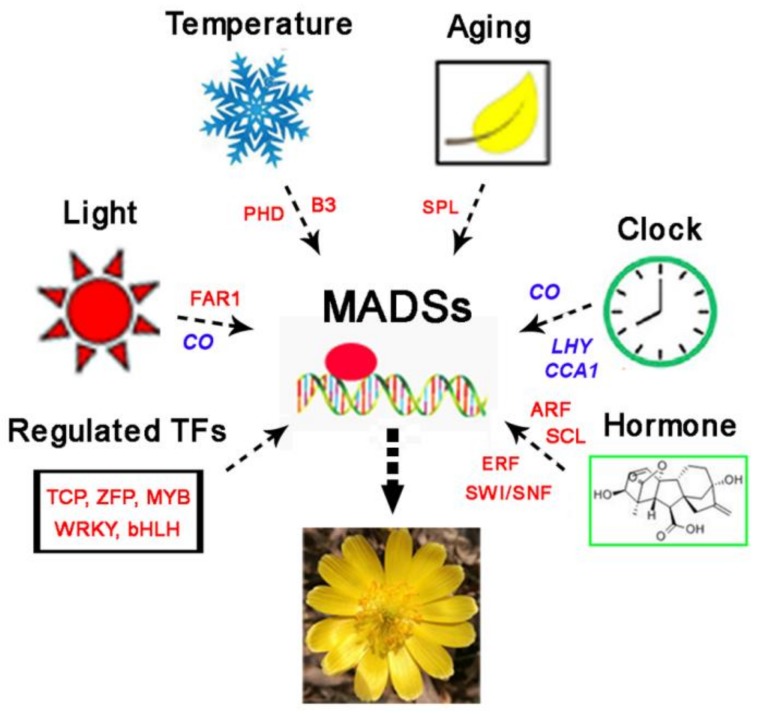
Hypothetical model of the regulation of flowering involving key transcriptional factors (TFs) in *A. amurensis*.

In conclusion, we used RNA-seq and iTRAQ to identify a large number of TFs, which may be involved in different regulatory pathways of *A. amurensis* flowering, including temperature, age, light, hormones, or other pathways (Figure 6). The identification and functional characterization of key regulatory TFs involved in these pathways may be helpful to reveal the genetic regulatory mechanisms that govern flowering in *A. amurensis* under low temperature conditions. However, the functions of these TFs require further characterization.

## Figures and Tables

**Table 1:** Number of functional annotations for all unigenes in public databases.

Values	Nr	InterPro	KOG	KEGG	Swiss-Prot	Nt	GO	Overall
Number	103,730	82,218	80,112	75,943	65,136	63,978	39,219	113,899
Percentage	39.39%	31.22%	30.42%	28.84%	24.73%	24.29%	14.89%	43.25%

**Table 2:** Summary of protein identification data.

Total Spectra	Spectra	Unique Spectra	Peptide	Unique Peptide	Protein
368,236	83,842	68,563	41,938	36,250	8427

## Supplementary Materials

The following are available online at https://www.mdpi.com/2073-4425/10/4/305/s1, Figure S1: Gene ontology (GO) functional classification of genes. The results are summarized in three main categories: biological processes (brown), cellular components (blue), and molecular functions (green). The *x*-axis indicates the number of genes, Figure S2: Species distribution of the top BLAST hits, Figure S3: The number of up- or down-regulated genes in FBD vs. YA, YA vs. VCA, VCA vs. EFS, EFS vs. FBS, and FBS vs. SFS in *A. amurensis*. We used a false discovery rate of ≤0.001 and an absolute log2 ratio of ≥1 as the threshold to determine significant differences in gene expression, Figure S4: Gene ontology (GO) functional classification of proteins. The results are summarized under three main categories: biological processes (brown), cellular components (blue) and molecular functions (green). The *x*-axis indicates the number of proteins, Table S1: 123 key transcription factors were identified in *A. amurensis* by combining RNA-seq and iTRAQ, Table S2: List of qPCR primers.

## Abbreviations

FAR1	Far-red-impaired response 1
PHY3	Far-red elongated hypocotyls 3
PHD	Plant homeodomain
ABI3VP1	ABA-insensitive 3/viviparous 1
SCL	Scarecrow-like
SWI/SNF	Switch/sucrose nonfermentable
ARF	Auxin response factor
ERF	Ethylene response factor
TCP	Teosinte branched 1/cycloidea/pcf
ZFP	Zinc finger protein
bHLH	Basic helix-loop-helix
MADS	Mcm 1/agamous/deficiens/srf
mTERF	Mitochondrial transcription termination factor
AP2-EREBPs	Apetala2-ethylene-responsive element binding proteins
CCA1	Circadian clock associated 1
LHY	Late elongated hypocotyl
TOC1	Timing of cab expressin 1
FT	Flowering locus T
TSF	Twin sister of FT
FLC	Flowering locus C
CO	Constans
SOC1	Suppressor of overexpression of CO 1
VRN2	Vernalization 2
VIN3	Vernalization insensitive 3
SVP	Short vegetative phase
GA	Gibberellin
GA20ox	Gibberellin 20 oxidase
SPL	Squamosa promoter binding protein-like
FUL	Fruitfull
PEP1	Perpetual flowering 1
