# Supplementary material for: Identification of Transcription Factors Involved in the Regulation of Flowering in Adonis Amurensis Through Combined RNA-seq Transcriptomics and iTRAQ Proteomics
Source: Genes (Basel). 2019 Apr 18;10(4):305. doi: 10.3390/genes10040305 (PMC6523232; doi:10.3390/genes10040305)
Supplement: Supplementary file 1 [file genes-10-00305-s001.zip › Supplementary Figures.pdf]

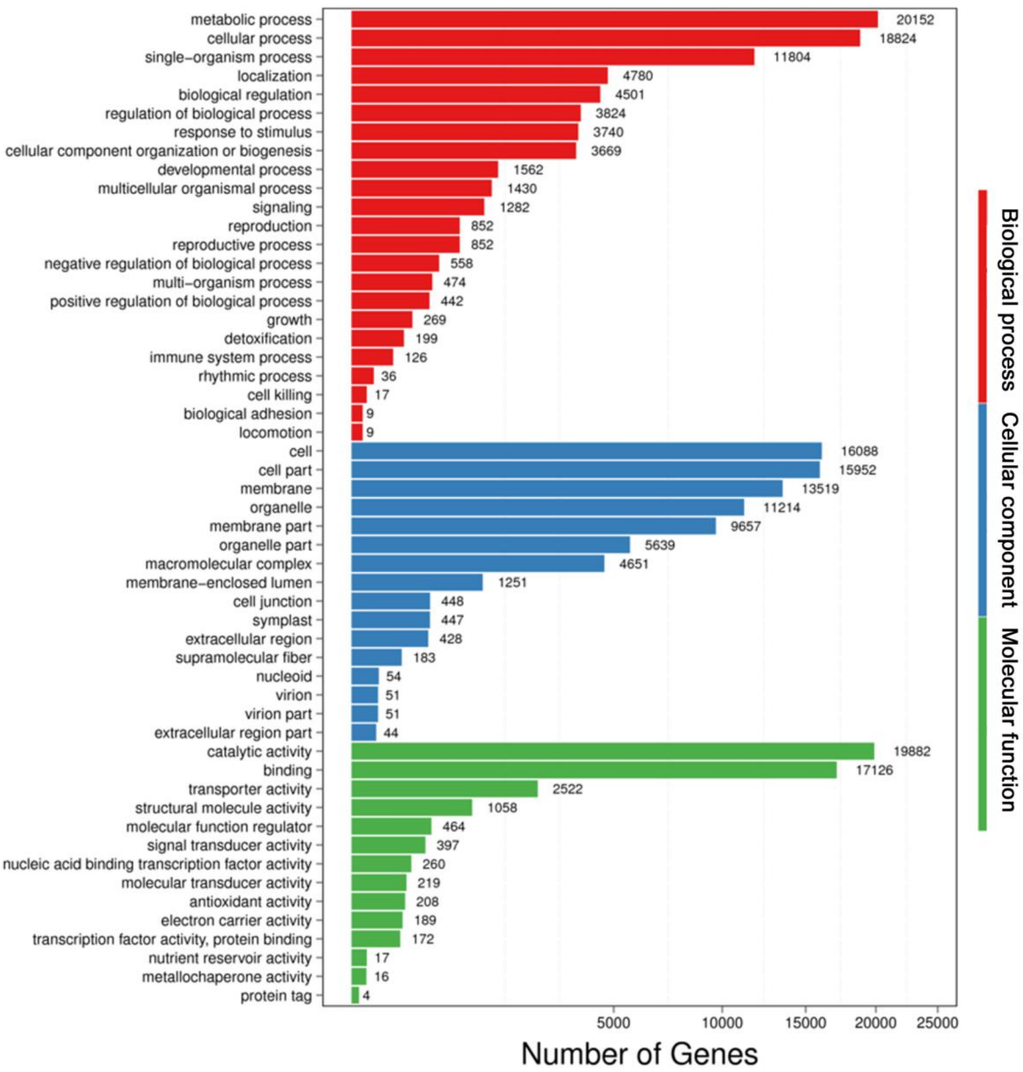

Figure S1. Gene ontology (GO) functional classification of genes. The results are summarized in three main categories: biological processes (brown), cellular components (blue), and molecular functions (green). The x-axis indicates the number of genes.

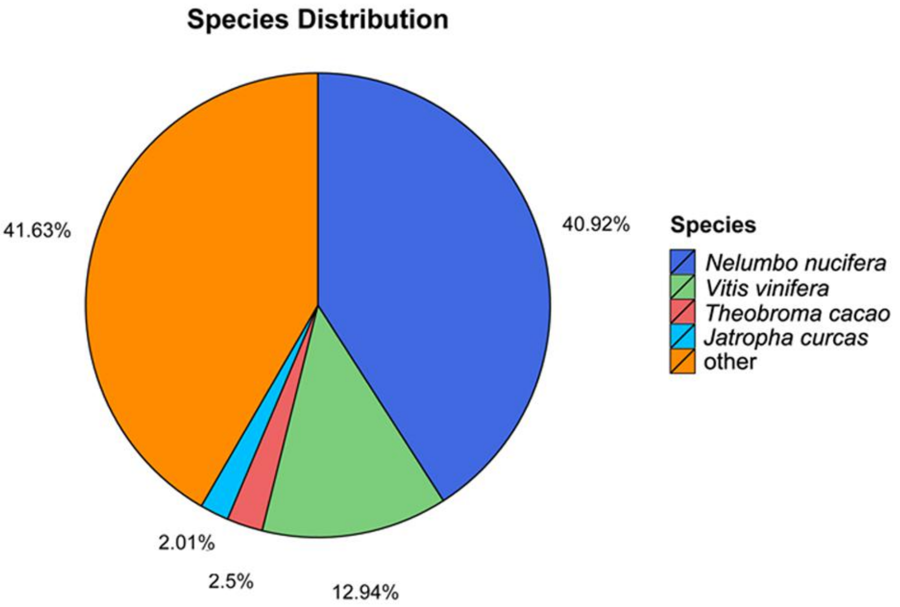

Figure S2. Species distribution of the top BLAST hits.

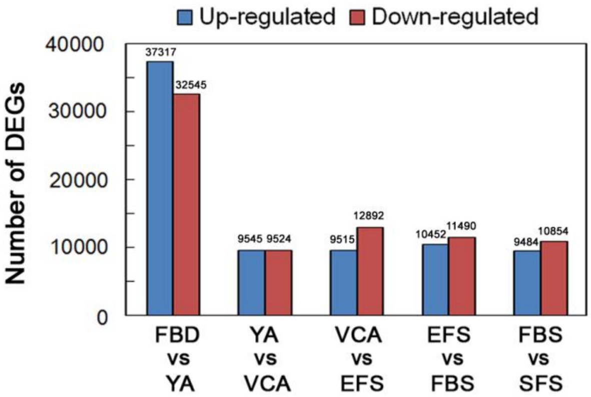

Figure S3. The number of up- or down-regulated genes in FBD vs YA, YA vs VCA, VCA vs EFS, EFS vs FBS, and FBS vs SFS in *A. amurensis*. We used a false discovery rate of ≤ 0.001 and an absolute log2 ratio of ≥1 as the threshold to determine significant differences in gene expression.

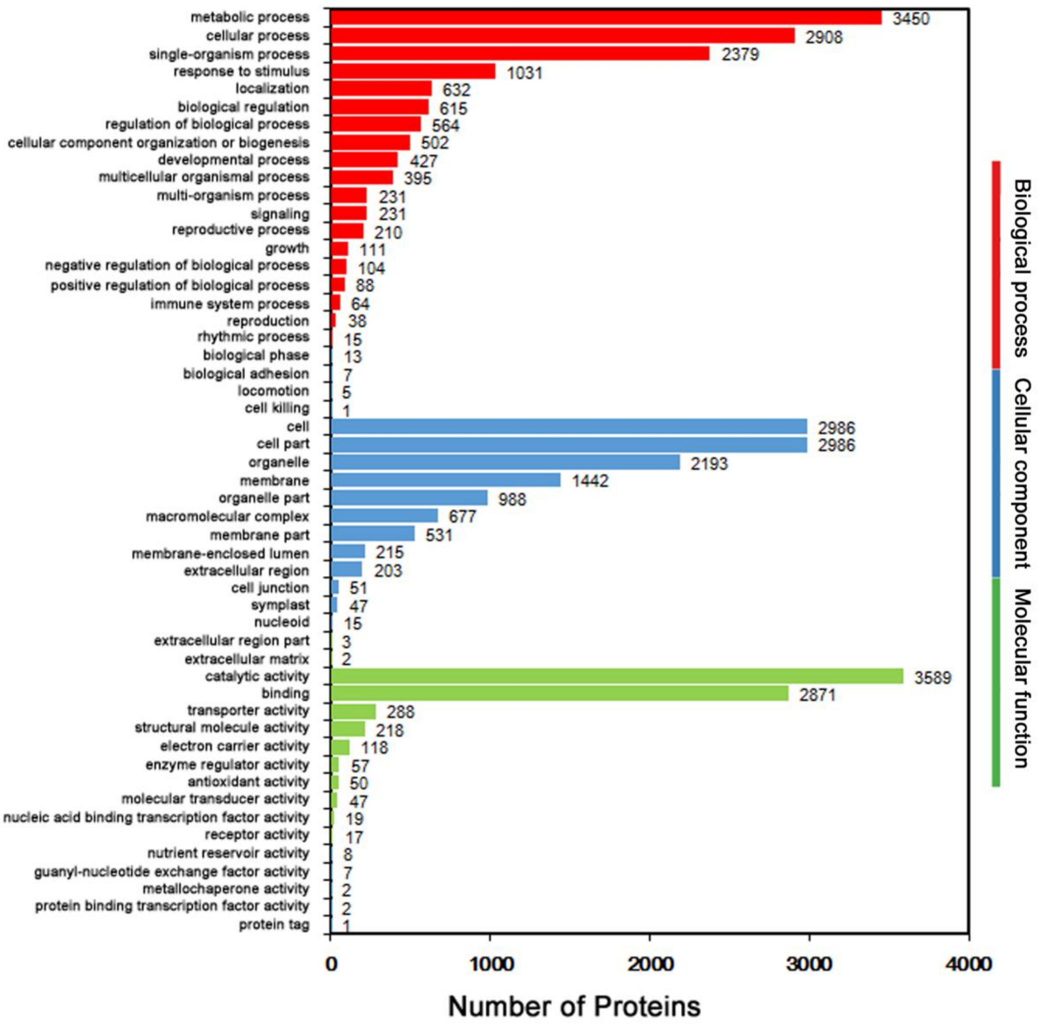

Figure S4. Gene ontology (GO) functional classification of proteins. The results are summarized under three main categories: biological processes (brown), cellular components (blue), and molecular functions (green). The *x*-axis indicates the number of proteins.
